# Sharpening Host Defenses during Infection: Proteases Cut to the Chase*

**DOI:** 10.1074/mcp.O116.066456

**Published:** 2017-02-08

**Authors:** Natalie C. Marshall, B. Brett Finlay, Christopher M. Overall

**Affiliations:** From the ‡Department of Microbiology & Immunology,; §Michael Smith Laboratories,; ¶Department of Biochemistry & Molecular Biology,; **Department of Oral Biological & Medical Sciences, Centre for Blood Research, Life Sciences Institute, University of British Columbia, Vancouver, British Columbia, Canada

## Abstract

The human immune system consists of an intricate network of tightly controlled pathways, where proteases are essential instigators and executioners at multiple levels. Invading microbial pathogens also encode proteases that have evolved to manipulate and dysregulate host proteins, including host proteases during the course of disease. The identification of pathogen proteases as well as their substrates and mechanisms of action have empowered significant developments in therapeutics for infectious diseases. Yet for many pathogens, there remains a great deal to be discovered. Recently, proteomic techniques have been developed that can identify proteolytically processed proteins across the proteome. These “degradomics” approaches can identify human substrates of microbial proteases during infection *in vivo* and expose the molecular-level changes that occur in the human proteome during infection as an operational network to develop hypotheses for further research as well as new therapeutics. This Perspective Article reviews how proteases are utilized during infection by both the human host and invading bacterial pathogens, including archetypal virulence-associated microbial proteases, such as the *Clostridia* spp. botulinum and tetanus neurotoxins. We highlight the potential knowledge that degradomics studies of host–pathogen interactions would uncover, as well as how degradomics has been successfully applied in similar contexts, including use with a viral protease. We review how microbial proteases have been targeted in current therapeutic approaches and how microbial proteases have shaped and even contributed to human therapeutics beyond infectious disease. Finally, we discuss how, moving forward, degradomics research can greatly contribute to our understanding of how microbial pathogens cause disease *in vivo* and lead to the identification of novel substrates *in vivo*, and the development of improved therapeutics to counter these pathogens.

Humans and their microbial pathogens have coevolved over time. Where many pathogenic bacteria and viruses were once major health threats—for example, those causing cholera and influenza—public health and medical advances have allowed us to combat these pathogens and significantly decrease infection-associated mortalities. In the developed world, many pathogens are no longer health threats. However, with the rise of antibiotic resistance, there is an imminent need to identify new drug targets. Therefore, we must further understand how pathogens cause disease and identify key proteins involved in the host response pathways.

Upon infection, multiple human immune regulatory pathways interface to integrate and selectively amplify cellular and systemic signals to protect against virulence processes, resolve infection, and achieve homeostasis once more. Defects in one pathway can destabilize the entire system, worsening disease over the course of infection and causing inflammatory and autoimmune diseases, as well as complications for patients suffering immunodeficiency diseases. Proteases are important regulators of signaling pathways in the initiation, progression, and resolution of inflammation, as well as in tissue and cellular function and extracellular matrix homeostasis yet are understudied in these contexts. Indeed, proteolytic processing—*i.e.* not degradation-to-completion but precise, highly efficient cleavage at one or two sites in a protein—has emerged as a key posttranslational mechanism to regulate signaling (1).

At 566 members, proteases are one of the largest human enzyme families, representing 1.7% of human genes, therefore larger than the kinase family (456 members) and second only to ubiquitin ligases (2). Proteolysis *in vivo* is more extensive than previously recognized: Remarkably, 44% of proteins in murine skin were processed and had amino (N) termini different to that annotated in UniProt, which increased to 60% during skin inflammation (3), and 64% of protein N termini in human erythrocytes were derived from proteolysis (4). Proteases are also essential regulatory molecules in immunity; they are responsible for the rapid mobilization and tethering of innate immune defenses, whether it be complement, antigen and MHC peptide processing, cytokine and chemokine activation or inactivation, and immune cell receptor activation pathways involving NF-κB (5–7).

In this Perspective Article, we review how proteolysis is utilized as an irreversible posttranslational modification (PTM) by microbes and the human host in host–pathogen interactions, focusing on bacterial pathogens. (Fungal protease virulence factors are reviewed elsewhere (8) and protease degradation has also been recently reviewed by Cohen-Kaplan *et al.* (9) and elsewhere.) We review how specialized proteomics approaches known as degradomics can significantly contribute to our understanding of the biological impacts of microbial and host proteases involved in disease, focusing on bacterial pathogens and including an illustrative example of a viral protease.

## 

### 

#### Precision Proteolysis: An Effective Posttranslational Regulator

Unlike most other PTMs, proteolysis is irreversible; whereas the activity of kinases can be reversed by phosphatases and that of ubiquitin ligases by deubiquitinating enzymes, protein religation is not kinetically favored. Therefore, instead of unregulated proteolysis, proteases are tightly controlled and can have extremely narrow substrate specificity, activation mechanisms, and effective inhibitors. This level of control allows proteases to act as highly specific and effective regulators of essential cellular functions. In addition to proteolysis as a protein degradation system, precision proteolysis is essential for many regulatory events, making proteolysis much more than degradation. During infection, successful invading pathogens must face rapid and irreversible proteolytic activation of immune defenses. Whereas the use of most PTMs to mobilize infectious or host defense processes can be defeated by countermeasures leading to their reversal, proteolysis represents a precise, rapid, and irreversible route to pathway modulation that avoids being easily defeated. Therefore, the proteolytic activation of essential pathway mediators is a clever strategy utilized by both microbe and host to evade defeat. Moreover, extracellular proteolysis represents one of the very last chances for a cell to posttranslationally modify secreted or circulating proteins, and so certain proteolytic systems in host defense can be viewed as standoff weapons.

Microbial proteases are also important factors to consider in infectious disease. However, it is position-precision proteolysis that is a hallmark of infection compared with host defense modulation. During infection, dysregulated protease activation can result in the processing of inflammatory mediators, altering their function *in vivo*, and ultimately causing excessive or nonresolving inflammation and damage to cells and tissue systems, leading to morbidity and sometimes mortality. Furthermore, microbial proteases as exotoxins play key roles in several human infectious diseases, and there still remains a wealth of information to be gained on precisely how and where these proteases affect host systems. On the other hand, this presents opportunities: Proteolytically processed cytokines and inflammatory/signaling mediators form a signature of infection-induced inflammation from which one can establish biomarkers for clinical diagnosis and to monitor treatment.

#### Host Defense: Immune Response under Proteolytic Control

The inflammatory response to infection and associated tissue damage involves a complex interplay of pathways and mediators, including Toll-like receptors, NF-κB-transcribed cytokine induction, innate and adaptive immune cell activation and recruitment, acute phase proteins, matrix metalloproteinases (MMPs), and the coagulation and complement proteolytic systems (7, 10, 11). Complement and coagulation cascades are archetypal cascades under proteolytic control and are increasingly recognized to be regulated by crosstalk from other proteases (3, 12) than originally described in the early classic studies. Cytokines, chemokines, and their receptors and binding proteins form pathways that guide tissue responses and inflammatory cell recruitment and activation (13–16); these proteins are increasingly recognized as being under the control of proteases (6), which can activate or inactivate cytokines, thereby modulating essential functions, without being predictable from transcript data alone. Furthermore, the relative balance and importance of each pathway and the degree of crosstalk during infection is unclear due to difficulties in identifying and quantifying low abundance mediators *in vivo*. This lack of knowledge hinders mechanistic understanding and clinical management of all infections as well as developing new diagnostic tests and drug development.

Quantifying host response pathways *in vivo* is not simple. mRNA levels do not always accurately reflect protein abundance (17), which itself may not reflect bioactivity, as bioactivity is often altered upon proteolytic processing and other PTMs. Chemokines are activated or inactivated by inflammatory cell MMPs (5, 18) and neutrophil elastase (19, 20). Surprisingly, proteomics analyses of protein N termini in inflamed skin revealed that complement cascade activation requires C1 inhibitor inactivation by MMP2 cleavage—*Mmp2*^−/−^ mice were significantly deficient in the ability to activate complement *in vivo* (3). Neutrophil MMP8 is required for chemotaxis toward lipopolysaccharide by proteolytic activation of murine CXCL5 and human interleukin-8 (21). More important though is the proteolytic inactivation of alpha-1 antitrypsin by MMP8 that allows elastase to also efficiently activate these chemokines *in vivo* (22). Macrophage-specific MMP12 both initiates and terminates neutrophil recruitment by chemokine cleavage (5, 23, 24), and MMP12 deficiency results in increased mortality in sepsis (25). MMP2 cleaves chemokine CCL7, converting it from a leukocyte chemoattractant to an antagonist, reducing cell infiltration, and dampening inflammation in murine peritonitis (18).

These studies highlight the need to move beyond the presence *versus* absence of proteins and to identify the relative amounts of intact *versus* cleaved forms of inflammatory mediators. These also form information-rich signatures that are needed to assimilate clinically relevant predictive knowledge to understand and treat infection and infection-induced inflammatory diseases. Ergo, *in vitro*, cell culture, and animal model analyses alone are not sufficient to understand the complexity of host responses actually at play *in vivo*. Systemwide approaches are needed and are now possible to identify and quantify proteolytic pathways *in vivo* in human tissue.

#### Studying the Interface: Proteomics of Infection

Infections and ensuing clinical syndromes arise from multiple stimuli that trigger concurrent signaling pathways, which are often entangled and further complicated as they can stimulate or antagonize each other. Hence, it is necessary to view infection-induced inflammation as a system, and unbiased systemwide approaches are required to understand the interplay of infection and inflammatory pathways to distinguish the particular host response to individual pathogens. However, the mechanistic understanding of infection-induced inflammation in humans has been hindered by the dearth of global analyses of inflamed or infected human tissue.

Proteomics is well suited for systemwide analysis of the complex interplay between signaling pathways and cell responses in infection and inflammation *in vivo*. Proteomics is a powerful tool to generate data and hypotheses that can be followed up by insightful mechanistic studies. In general, proteomics applications have greatly contributed to studying pathogens and host–pathogen interactions (reviewed by (26–28)). To establish clinically relevant host or microbial protease substrates and interactions in inflammation, proteolysis should be studied in tissue, where cells are in their natural microenvironments, proteases and inhibitors are at biologically relevant concentrations, and cells respond to natural environmental cues. By assessing net proteolytic activity in tissue, the proteolytic signature obtained can be used to identify cleaved proteins in signaling networks, and these events can be ranked for their contribution to relevant bacterial host response pathways. For example, stable isotope labeling by amino acids in cell culture and quantitative proteomics have enabled the identification of virulence factors (27) and the cataloguing of the virulence-associated secreted protein repertoires, known as secretomes, of many pathogens (29–34); enrichment techniques and mass spectrometry (MS) have enabled the identification of virulence factor substrates and host binding partners (35–37); shotgun proteomics has allowed the characterization of the host cellular response to infection at a global level (38–40), and proteomics techniques specific for various PTMs, including phosphorylation, have further characterized bacterial regulation (41) and the host cellular response, as well as identifying mechanisms of virulence factors (42, 43).

#### Degradomics: Quantifying Intact and Neo-N and -C Protein Termini In Vivo

Studies analyzing global protein and protease networks *in vivo* in infected and inflamed human tissues have, until recently, proven intractable: Proteolytic cleavage is opaque to conventional proteomics, which therefore limits functional insight. In conventional proteomics, 100,000s of tryptic peptides in a proteome that is dominated by abundant proteins are analyzed by MS, thereby further burying natural or protease-generated neo termini, especially for rare or low abundance mediators like chemokines. Unbiased system-level approaches to proteolytic regulation constitute the field of degradomics (44). Protease degradomics is the identification of proteases and their full set of substrates on a global, proteomewide scale (44). Degradomics aims to study the protein terminal proteomes, or “terminomes,” *i.e.* to identify the exact site and sequence of the protein amino and carboxyl (C) termini of intact substrates and thereby identify exact sites of proteolysis.

Two of the leading terminomics techniques are terminal amine isotopic labeling of substrates (TAILS) (45, 46) and combined fractional diagonal chromatography (47, 48); both involve the selective negative enrichment of terminal peptides from a digested proteome and the use of MS and bioinformatics to identify: the terminal peptide sequence, the protein that the terminal peptide originated from, and accordingly the position of the terminal peptide within the mature protein. Other degradomics techniques include the 1D gel-based approaches Protein Topography and Migration Analysis Platform and Global Analyzer of SILAC-Derived Substrates of Proteolysis, as well as the subtiligase-catalyzed biotinylation of protein N termini for subsequent capture and enrichment (49–51).

Degradomics has been successfully applied to many studies (reviewed by Eckhard *et al.* (52)), as it enables: characterization of protease specificity (53); identification of zymogen and signal peptide cleavage sites (54); identification of novel protease substrates (55–57), including those involved in lymphocyte antigen receptor activation (7); and characterization of downstream effects of particular proteolytic events (23, 57). Accordingly, degradomics has been applied to study topics as diverse as apoptotic cell death (58, 59), arthritis (12), protease–protease crosstalk (22), biomarker identification (60), and validation or correction of genome annotations (61). For example, we identified >2,400 proteins in inflamed murine skin: Of the protease-generated neo-N-terminal peptides, 127 were elevated >7-fold *in vivo*, forming a proteolytic signature that distinguished inflamed from normal skin. With this signature, we identified novel and unexpected roles for protease crosstalk in activating complement and releasing the inflammatory mediator bradykinin in the inflamed tissues of wild-type mice but not in MMP2 knock-out mice (3). In another *in vivo* study, Bellac *et al.* (12) analyzed murine arthritic joints at different time points by an effective eightplex iTRAQ TAILS approach. Here, it was found that the macrophage MMP12 was host protective by cleaving and inactivating multiple components of the complement pathway, including the potent chemoattractant factors C3a and C5a. Proteolytic signatures of cleaved proteins with altered activity can also form biomarkers of subclinical *versus* clinical infection *versus* advanced disease stage, which can be mechanistically informative and followed up for novel insights into the regulation of human host defense pathways and their crosstalk. In the future, we anticipate that proteolytically modified proteins will be translated as new clinical tests to determine the risk and imminence of infection-induced pathology and to identify candidate drug targets for managing disease.

The most widely adopted terminomics approach is terminal amine isotopic labeling of substrates (TAILS): N-TAILS for neo-N termini (45, 46) and C-TAILS for neo-C termini (62). The key to this technique is labeling α-amines at the protein level, thereby labeling and chemically blocking mature protein N termini and neo-N termini resulting from proteolysis. Labeled N-terminal α-amines are strong evidence for their presence *in vivo* and their proteolytic generation in the biological sample and are therefore considered true positives. However, the use of protease inhibitors at sample collection and careful handling is essential to prevent post-harvest cleavage by proteases still active in the sample. Nonetheless, background proteolysis in the sample or inadvertently through sample handling is identifiable from labeled peptides having isotopic ratios centered on 1:1, whereas the protease-exposed sample will have high isotope ratios (*i.e.* +protease sample/-protease sample) for true positive cleaved peptides. Later in sample preparation, internal tryptic peptides display newly exposed free α-amines arising from trypsin cleavage that were not exposed and blocked earlier in the procedure (45, 46). These unblocked tryptic peptides are depleted from the sample using a polyaldehyde polymer (http://www.flintbox.com/public/project/1948/) that was designed to bind to free α-amines under reductive conditions, leaving the original sample N-terminome unbound and easily recoverable. To improve the identification of protein C termini, which lack positive charges in trypsin-digested samples, an archaeal protease, LysargiNase, can be used to mirror trypsin specificity and vastly improve C-terminal peptide identification (63). TAILS is amendable to many different protein labeling techniques, including dimethylation, stable isotope labeling by amino acids in cell culture, iTRAQ, and most recently isobaric tandem mass tags (TMT) (46, 64). These labeling techniques allow up to 10 samples to be analyzed simultaneously, reducing experimental error due to technical variability.

Although metabolic labeling approaches such as stable isotope labeling by amino acids in cell culture are the gold standard in quantitative proteomics, they are practically impossible in humans. Therefore, 10-plex isobaric TMT is the state-of-the-art for quantitative analysis of human infection and tissues. Of relevance to host antimicrobial immune responses, we utilized 10-plex TMT-TAILS to analyze B cells from the only reported *MALT1* paracaspase-deficient living patient in the world *versus* cells from healthy heterozygous family members. This enabled a global examination of MALT1 cleavages in an intact human cell system that directly linked MALT1 paracaspase activity to cessation and resetting of NF-κB signaling in human and murine B and T cells (7), contrary to the view at the time that MALT1 substrates activated signaling. Thus, in B cells it was found that nonproteolytic scaffolding by MALT1 initiates signaling by NF-κB, but MALT1 then terminates signaling by cleaving HOIL1 in the linear ubiquitin assembly complex to turn off linear ubiquitination that is essential for B and T cell receptor microcluster formation, signal transduction, and NF-κB activation. The absence of linear ubiquitinated proteins for ∼2 h rendered the cells refractory to ongoing B and T cell receptor stimulation, thus downregulating overactivation of the antigen receptors.

Despite the breadth of prior degradomics applications, relatively few have studied pathogens and host–pathogen interactions in infection *in vivo*. Degradomics can identify substrates of pathogen-encoded proteases, both intracellular and secreted. Furthermore, for pathogens that do not encode known proteases, degradomics of infected host cells would be valuable to glean mechanistic information on how the host cell proteome and global proteolysis change during infection, including how host processes are impacted during infection. This approach could identify mechanistic causes of disease symptoms and map the pathways behind it.

#### Microbial Offense: Protease Manipulation during Infection

Many pathogens manipulate proteases as a virulence strategy, ultimately causing injury to host tissues or evasion from the host immune system. Indeed, many bacterial proteases play a direct and substantial role in disease. For example, it has long been known that *Clostridia* spp. secrete potent collagenases that aid infection by disrupting tissue barriers while providing amino acids as a carbon source (65). Furthermore, botulism and tetanus are caused by the botulinum and tetanus neurotoxins (BoNT; TeNT) of the Gram-positive bacteria *Clostridium botulinum* and *Clostridium tetani*. Both toxins are zinc metalloproteases that cleave proteins involved in neurotransmitter release. BoNT prevents the release of neurotransmitters, resulting in flaccid paralysis, and TeNT prevents the release of inhibitory neurotransmitters, resulting in spastic paralysis (66, 67). BoNT and TeNT are required and sufficient to cause the symptomatic paralysis observed in these two diseases and are some of the most lethal compounds known.

The Gram-negative pathogen *Yersinia pestis* is best known as the causative agent of bubonic plague. Unlike closely related *Yersinia* species, *Y. pestis* encodes Pla: a surface aspartic protease with adhesive and invasive properties (68). Pla cleaves plasminogen, thereby activating plasmin (69), which breaks down many plasma proteins, including fibrin clots. The primary inhibitor of plasmin, α_2_-antiplasmin, is also cleaved and inactivated by Pla (70), resulting in unregulated plasmin activity and damage to host tissues. Evolutionary acquisition and optimization of *pla* increased this pathogen's ability to invade and disseminate within the host, leading to speculation that the Pla protease was an essential factor enabling *Y. pestis* to cause bubonic plague and to spread to pandemic levels during the Black Death in the 1300s (68). Indeed, when the *pla* gene was experimentally inactivated, the *Y. pestis* lethal dose increased by >1 million-fold in a mouse model (69).

Some bacterial pathogens encode proteases that assist with escape from the host immune system: their largest and most imminent threat during infection. For example, many pathogens that infect mucosal surfaces encode proteases that cleave immunoglobulin A1 (IgA1), including *Neisseria meningitidis* and *Neisseria gonorrhoeae*, which cause human meningitis and sexually transmitted infections (70). IgA1 proteases separate the pathogen-recognition (Fab) and host signaling (Fc) components of the antibody, thereby severing communication with host defense cells. This also leaves pathogens coated with cleaved Fab fragments and camouflaged from the immune system. IgA1 proteases disable this important defense immune molecule, allowing for direct escape of the invading pathogen from host immunity. Different pathogens have different mechanisms for tackling the immune system. *Serratia marcescens*—which causes various hospital-acquired infections—tackles immune cells directly with the metalloprotease serralysin. Serralysin is secreted and bound by a host protease inhibitor in the plasma, α-2-macroglobulin (α_2_M). Serralysin-α_2_M complexes are internalized by fibroblasts and macrophages, resulting in targeted cytotoxicity within these immune cells (71), and thereby allowing this pathogen to attack immune cells directly, specifically, and from within.

Bacterial pathogens can also cause disease without providing their own specialized proteases; many target and disrupt *host* proteases, instead. For example, many pathogens activate plasminogen, including: *Y. pestis*, *N. meningitidis, Pseudomonas aeruginosa*, group A/C/G streptococci, *Staphylococcus aureus*, and *Escherichia coli*. Some pathogens make use of host proteases, such as *Salmonella enterica* serovar Typhimurium, which infects the human gut, causing neutrophil recruitment to the gut lumen and secretion of neutrophil elastase. Elastase causes shifts in the composition of the gut microbiome and ultimately creates a more favorable environment for *Salmonella* colonization and the initiation of disease (72). Rather than activating host proteases, some bacterial pathogens directly inhibit host proteases. Enteropathogenic *E. coli* delivers its virulence factors, including NleF, into intestinal epithelial cells. Within the host cell cytoplasm, NleF directly binds to and blocks the activity of host caspases-4, -8, and -9 (73).

These examples demonstrate the broad and important roles that bacterial proteases and bacterial manipulation of host proteases have in infectious diseases. These paradigms of bacterial pathogenesis have all been established in the field using microbiology, biochemistry, and molecular biology techniques and notably without 'omics techniques. In recent decades, 'omics techniques have made a substantial impact in understanding virulence mechanisms and host–pathogen interactions. Unlike other 'omics techniques, degradomics is sensitive to alterations made by proteases. Its success in other applications shows that degradomics techniques can identify substrates of a protease of interest, the specific sites of proteolysis, and motifs to identify protease specificity. The importance of proteases in host–pathogen interactions highlights both the need for such knowledge as well as its potential impact.

#### Microbial Degradomics

More recently, degradomics has been applied to study bacteria, recently reviewed by Berry *et al.* (74). The degradomes of 10 eubacterial species (75–83) and at least three archaeal species (84–86) have been studied. These studies include two human pathogens (*N. meningitidis* and *Coxiella burnetii*, which causes Q fever) but are not limited to pathogens, also including model bacterial organisms. These recent studies of bacterial degradomes have helped to revise and improve bacterial genome annotations, identify signal peptide locations (75), identify *in vivo* specificity of individual proteases, study the prevalence of initiator methionine removal across a eubacterial proteome (77), the effects of N-terminal acetylation across an archaeal proteome (85), examine the effects of antibacterial treatment (87), and identify proteins involved in antibiotic resistance (88).

These degradomics studies have contributed to our understanding of bacterial regulation, translation, secretion, and proteolysis: processes that are important for many bacterial traits and functions. Virulence is also tightly and specifically regulated at many of these levels, from gene regulation to protein secretion and proteolytic processing. Therefore, degradomics is also valuable to study how pathogens infect and cause disease and to identify proteins and proteoforms that pathogens deliver to the host environment.

#### Degradomics of Infection: Current Status and Possibilities

Just as we can learn about pathogens by their study in isolation, we can learn about virulence and disease by studying pathogens and their host cells together. The application of degradomics to study the bacterium–host interface could generate a wealth of knowledge on infectious diseases, yet, to our knowledge, no applications have been reported. For example, host substrates of microbial proteases could be identified through simple *in vitro* degradomics studies in cell culture models. Furthermore, whether or not pathogens employ proteases as virulence factors, virulence processes can substantially affect infected cells. Therefore, by studying the impact of infection on the host proteome, global changes across the entire cell may be viewed more comprehensively. Moreover, these studies will be inherently less biased than the classical approach of studying a targeted subset of host proteins or pathways and will require less *a priori* knowledge of pathogenic mechanisms. This is particularly valuable for the study of nonmodel organisms and pathogens without tractable genomes, such as *Chlamydia* spp., where the lack of molecular tools is a barrier to further research.

Degradomics studies of infected cells would cast light upon host cell pathways involved during infection. For example, degradomics has been utilized to identify human substrates of viral proteases and to characterize unexpected roles of human proteases in antiviral immune defense. Marchant *et al.* used TAILS to identify novel substrates of human macrophage-specific MMP12, which is essential for interferon (IFN)^1^ α secretion and thus for effective antiviral defense; in an otherwise nonlethal viral infection, mice lacking MMP12 have a fatality rate >30% (89). While MMPs are typically associated with extracellular matrix degradation, compelling evidence was found for macrophage MMP12 in the nucleus of cells infected with coxsackievirus B3 and respiratory syncytial virus. When *Mmp12*^−/−^ cell lysates were incubated with active MMP12 and analyzed using TAILS, 328 new cellular substrates were identified. ChIP-SEQ and transcription reporter assays also identified a cohort of genes that were transcriptionally up-regulated upon MMP12 promoter binding or down-regulated upon exon binding, including 177 MMP12 substrates identified by TAILS, including IFNα. This study revealed an unexpected story of MMP12 in antiviral defense: Upon secretion by macrophages, MMP12 translocates to infected cell nuclei, where it binds to DNA. MMP12 binding to the IκBα promoter is essential for transcriptional up-regulation of IκBα, which is essential for IFNα secretion and antiviral immunity. Outside the cell, MMP12 cleavage also forms a feedback loop to down-regulate systemic IFNα by degradation, reducing the toxic and systemic actions of prolonged, elevated IFNα (89). Thus, a host defense protease both initiated IFNα secretion and then cleared the cytokine from the circulation several days later.

More recently, TAILS was applied to identify host substrates of viral proteases *in vitro*. Jagdeo *et al.* incubated poliovirus and coxsackievirus 3C proteases with a human cell lysate and performed TAILS to identify known 3C substrates, as well as novel substrates, one of which was validated: heterogeneous nuclear ribonucleoprotein M (90). Heterogeneous nuclear ribonucleoprotein M was shown to be cleaved by both viral 3C proteases, resulting in a nuclear to cytoplasmic relocalization, and was important for optimal poliovirus and coxsackievirus infection.

These studies demonstrate the value of degradomics to study the host–pathogen interface and the potential of degradomics techniques to study the pathogen effects and cellular response to infection, particularly to generate testable hypotheses for the mechanisms of virulence and host immunity.

#### Getting Ahead of the Pathogen by Undermining Protease-Mediated Virulence

Proteases have a well-established impact on human health in infectious diseases. Many microbial proteases are therefore valid therapeutic targets. As knowledge accumulates on the mechanism of action of bacterial and viral proteases, the pharmaceutical industry has become increasingly empowered to develop therapeutics to tackle proteases and so treat infection. Microbial proteases have been successfully subverted or inactivated in key pathogens with great effect. Some of the best examples are clinically approved inhibitors targeting the hepatitis C virus NS3/4A protease (91) and the human immunodeficiency virus (HIV)-1 protease, which is required for viral replication and spread (reviewed by De Clercq (92) and Patick and Potts (93)). Specific inhibitors have also been developed against bacterial proteases, including botulinum, tetanus, and anthrax toxin proteases from *Clostridium* spp. and *Bacillus anthracis* (94, 95); these proteases have been successfully targeted by neutralizing antibodies, resulting in inactivation by passive immunization (96). Although only a small number of protease inhibitors are currently available to treat bacterial infections, several studies demonstrate the feasibility of treating other microbial diseases by targeting their proteases. For example, the Gram-positive bacterium that causes necrotizing fasciitis and toxic shock syndrome, *Streptococcus pyogenes*, encodes several virulence-associated proteases that, when deleted, render the pathogen less virulent and easier to overcome by the host immune system. The *S. pyogenes* C5a peptidase cleaves complement factor C5a at the site of leukocyte receptor binding, preventing leukocyte influx to the site of infection and ultimately enabling the pathogen to avoid phagocytosis. However, when the C5a peptidase gene is removed, *S. pyogenes* infection is successfully cleared (97), thus these gene products represent promising candidate drug targets.

Similarly, the Gram-negative opportunistic pathogen *P. aeruginosa* encodes multiple proteases that are important for infection, such as aeruginolysin, elastase, protease IV, and two endopeptidases. In a mouse model of burn trauma, *P. aeruginosa* infections are significantly less severe in strains lacking these proteases (98) or when a protease inhibitor is injected into host tissues during infection (99). These studies demonstrate the susceptibility of pathogens lacking their virulence-associated proteases and their potential treatment with protease inhibitors. Ironically, these approaches mirror Koch's postulates—a set of criteria established by bacteriologist Robert Koch to determine the causation between bacterial species and disease (100, 101)—to highlight the importance of the proteases themselves in the course of the disease, as well as the feasibility of targeting them to effectively resolve bacterial diseases.

Specific inhibitors of bacterial proteases show potential to improve upon the current standard of care and increase the longevity of current antimicrobial therapeutics. In the era of increasing antibiotic resistance, therapeutic approaches that apply minimal selective pressure for resistance are increasingly sought-after, such as those targeting specific virulence mechanisms. Rather than killing the pathogen by targeting pathways essential for life, therapeutics can instead be developed to *disarm* the pathogen by targeting and neutralizing specific components essential for virulence, thereby decreasing the selective pressure on the pathogen (96, 102) and increasing the length of therapeutic effectiveness. This approach is particularly valuable for spore-forming pathogens, such as those producing the botulinum, tetanus, and anthrax toxin proteases. When these bacteria sense stress, such as antibiotic treatment, they sporulate to resist death and later germinate and revive, making such bacteria a challenging target for effective antibacterial treatment. Furthermore, in the case of the botulinum, tetanus, and anthrax toxins, which are sufficient to cause disease (103–105), even when antibacterials are effective, the toxins can still be present in the host's system in such high quantities that may still lead to death. Therefore, the directed and specific targeting of these proteases is a valuable treatment option.

These current botulinum and tetanus antitoxins are neutralizing antibodies, which are effective for extracellular toxin that is not currently bound to its host substrate. Therefore, specific, cell-permeable, small molecule protease inhibitors would be even more effective, and small molecules would be able to prevent or resolve symptoms that other treatments cannot, either by directly inhibiting any accumulated toxin or by altering virulence gene expression. This approach has been shown to be effective with *S. pyogenes*, where small molecules effectively disrupted streptokinase gene expression, thereby disrupting virulence (106). When those small molecules were administered, phagocytosis and killing was enhanced (106).

#### Know Your Enemies: Using Their Abilities in Disease to Our Benefit in Health

Paradoxically, potent bacterial proteases can also be used to our advantage: Whereas bacterial proteases are extremely effective agents against essential human systems, they can also be used as clinical treatments for some human diseases. For example, many bacteria produce molecules against bacterial competitors to acquire or maintain an advantage within an ecological niche. *Staphylococcus simulans* produces the metalloprotease lysostaphin, which cleaves and compromises staphylococcal cell walls and is highly effective against *Staphylococcus aureus* (107–110). Lysostaphin has also been shown to be effective against methicillin-resistant *S. aureus* clinical isolates (111) and has shown promise in animal studies (107, 112, 113). Likewise, *Streptococcus zooepidemicus* produces a lysostaphin-like protease effective against *Streptococcus pyogenes* (114). The botulinum toxin protease (or, BOTOX®) has been developed for both clinical and esthetic use (115), including treatment for cerebral palsy (116), chronic migraines (117), and the particularly serious aliment, facial wrinkles.

Similarly, bacterial proteins that are known to affect human proteases have been effectively used against natural human target(s) as clinical treatments for other human diseases. For example, *Staphylococcus*-produced staphylokinase (118) and *Streptococcus*-produced streptokinase (119): Neither are known to possess enzymatic activity on their own, but they are effective thrombolytic agents and are administered as a treatment following heart attack and pulmonary embolism (119).

#### Perspectives

In infectious disease the distinction between causation and association is key. The first tools to address this were Koch's postulates, a set of three criteria proposed by Robert Koch in the late 1800s to assess a causative link between the presence of bacterial species during disease and the cause of disease itself (100, 101). Koch's postulates have weaknesses and have since been reviewed (120) and ultimately supplanted with more rigorous standards, such as the Bradford Hill criteria, also known as Hill's criteria for causation (121). Hill's criteria help evaluate the strength of an association between a pathogen or compound and a particular disease. Hill's nine criteria for causation are: strength, consistency, specificity, temporality, biological gradient, plausibility, coherence, experiment, and analogy. It has been argued that 'omics tools allow for “a systems approach to extract causation from association” (122). Indeed, 'omics tools do provide some advantage in examining Hill's criteria. With proper experimental design, 'omics datasets can be mined for the strength and consistency of the association within the same datasets and between technical replicates. Due to the advances of multiplexing several experimental conditions within a single mass spectrometry run (7), and the alternate possibility of employing metabolic labeling strategies, such as SILAC, modern degradomics techniques allow for the minimization of technical variability while considering relevant controls (*e.g.* protease-null, uninfected, noncleavable substrate). Similarly, unbiased tools such as 'omics tools provide a wealth of data that can lead to multiple approaches to more easily identify the plausibility of associations. Thus, future research in infectious diseases would be substantially increased by the new insights gained by further endeavors using degradomics. These applications may shed light on the global effects of microbial proteases, including identifying their full substrate repertoire and cleavage motifs, and would be valuable to study the dysregulation of host pathways during infection that are significant during disease. And finally, in conducting 'omics work with the appropriate biological question and experimental design, one can satisfy the experiment and the biological gradient (*e.g.* higher infecting dose, higher protease concentration), including the temporality of the association (*e.g.* early and late time points). It is therefore reasonable that techniques like degradomics can, at the very least, serve as a more high-throughput way by which to address Hill's criteria.

Proteolytic signatures of infection-driven inflammation and intracellular host responses have the potential to provide global overviews of these pathways in humans and can be established by the use of degradomics. Proteolytic signatures can also be used to establish the clinical and mechanistic importance of regulatory proteolytic processes in the human host response and the crosstalk occurring between different inflammatory pathways operating *in vivo* in infection. Such studies offer great potential to generate novel mechanistic and clinically relevant knowledge from pathway analyses and proteolytic signatures on new protease and cleaved substrate drug targets in important human infections. Cleaved proteolytic fragments of these proteins offer a new route for developing predictive and mechanistically informative biomarkers for translation to the clinic for early and more precise diagnosis of the course of infection (123, 124). This knowledge, combined with much needed new diagnostic tests, will improve diagnosis and selection of patients for active and beneficial clinical intervention, as well as for future pandemic preparedness.

## Footnotes

^1^ The abbreviations used are:

IFN: interferon
α_2_M: α-2-macroglobulin
BoNT: botulinum neurotoxin
HIV: human immunodeficiency virus
IgA1: immunoglobulin A1
MMP: matrix metalloproteinase
MS: mass spectrometry
NF-κB: nuclear factor kappa B
MALT1: mucosa-associated lymphoid tissue lymphoma translocation protein 1
ChIP-Seq: chromatin immunoprecipitation sequencing
IκBα: NF-κB inhibitor alpha
PTM: posttranslational modification
spp.: species
TAILS: terminal amine isotopic labeling of substrates
TeNT: tetanus neurotoxin
TMT: tandem mass tag.
